# Supplementary material for: The Executive Functioning Paradox in Substance Use Disorders
Source: Biomedicines. 2022 Oct 28;10(11):2728. doi: 10.3390/biomedicines10112728 (PMC9687962; doi:10.3390/biomedicines10112728)
Supplement: Supplementary file 1 [file biomedicines-10-02728-s001.zip › biomedicines-1949966-supplementary.pdf]

Table S1. Description of the 512 Regions of Interest as described by Yeo Dictionary Atlas.

Component	Difumo_names	Yeo_networks17	GM	WM	CSF
1	Superior occipital sulcus inferior LH	DorsAttnA	0.575880204	0.346929161	0.077030615
2	Superior occipital gyrus	VisCent	0.472506536	0.495570263	0.031931908
3	Insula antero-inferior	SalVentAttnA	0.720733158	0.054171999	0.225070424
4	Middle frontal gyrus middle RH	SalVentAttnB	0.64341873	0.245515795	0.06801427
5	Calcarine cortex posterior LH	VisCent	0.553855658	0.414815055	0.031008928
6	Lateral fissure posterior ramus RH	SalVentAttnA	0.452173118	0.511383637	0.036402382
7	Superior occipital gyrus superior LH	DorsAttnA	0.306230862	0.648622337	0.044662029
8	Angular gyrus inferior LH	TempPar	0.727386807	0.199754034	0.072504452
9	Calcarine sulcus anterior LH	VisPeri	0.622813828	0.325256655	0.051944418
10	Frontal pole inferior RH	ContB	0.661148398	0.194421023	0.095687053
11	Midbrain superior	No network found	0.671035561	0.159968349	0.166200084
12	Occipitotemporal gyrus mid-posterior RH	DorsAttnA	0.722065697	0.236968357	0.040886573
13	Insula postero-inferior medial	SomMotB	0.668649838	0.307668693	0.023684975
14	Cerebellum VI RH	No network found	0.596015348	0.396682033	0.007307209
15	Fusiform gyrus anterior LH	DorsAttnA	0.830749839	0.059461896	0.109786969
16	Cerebellum Crus I anterior RH	No network found	0.59737708	0.370113979	0.030346592
17	Supramarginal gyrus antero-superior LH	DorsAttnB	0.494749561	0.457885497	0.045460359
18	Angular sulcus superior LH	DefaultB	0.559949202	0.39108251	0.040272585
19	Paracingulate sulcus LH	SalVentAttnB	0.45623692	0.520651682	0.023111699
20	Superior frontal gyrus superior	SomMotA	0.592332496	0.283522638	0.103399448
21	Pars triangularis LH	DefaultB	0.655862037	0.07753695	0.139295998
22	Superior longitudinal fasciculus II LH	No network found	0.032083448	0.967199838	0.000715201
23	Corona radiata antero-superior	No network found	0.0195889	0.979745385	0.000686319
24	Dorsomedial prefrontal cortex antero-superior LH	DefaultB	0.640048273	0.229680642	0.105655437
25	Superior longitudinal fasciculus III anterior LH	No network found	0.042096041	0.957843369	4.96042E-05
26	Cuneus antero-inferior	VisPeri	0.611854399	0.288172329	0.099982467
27	Anterior vertical ramus of the lateral fissure LH	DefaultB	0.66349062	0.261661676	0.073778579
28	Corpus callosum isthmus anterior	No network found	0.193872689	0.421464625	0.384676341
29	Internal capsule posterior limb RH	No network found	0.247868556	0.749999699	0.002128016
30	Cingulate gyrus middle inferior	ContC	0.613005096	0.29075291	0.096239725
31	Caudate RH	No network found	0.578796166	0.409584503	0.01163335
32	Superior frontal sulcus anterior RH	ContB	0.468779704	0.496426923	0.03359863
33	Pars orbitalis RH	DefaultB	0.655764398	0.275065854	0.05382516

Table S1. Description of the 512 Regions of Interest as described by Yeo Dictionary Atlas.

34	Cerebellum VIIb	No network found	0.781835485	0.114307576	0.098836578
35	Intraparietal sulcus anterior LH	DorsAttnB	0.560249094	0.152479695	0.211712088
36	Superior longitudinal fasciculus I posterior RH	No network found	0.030552203	0.969327053	0.00012589
37	Superior longitudinal fasciculus II middle RH	No network found	0.0181254	0.981858052	1.35373E-05
38	Angular gyrus postero-inferior RH	DefaultC	0.716772083	0.208349471	0.07477112
39	Cerebrospinal fluid (between middle frontal gyrus and skull)	DefaultB	0.401517489	0.016921212	0.114356378
40	Cerebellum III anterior	DefaultC	0.498736098	0.132502712	0.313075873
41	Central sulcus middle RH	SomMotA	0.509983045	0.346122125	0.142123435
42	Cerebrospinal fluid (between interhemispheric fissure and superior frontal gyrus superio)	SalVentAttnA	0.44398159	0.034462534	0.218732307
43	Superior frontal gyrus superior RH	DefaultB	0.659105575	0.268342523	0.061915794
44	Frontal pole superior RH	DefaultA	0.529705764	0.091767189	0.095294293
45	Postcentral sulcus LH	DorsAttnB	0.655613935	0.253170918	0.091178842
46	Frontomarginal gyrus	DefaultA	0.449605314	0.514653437	0.032644029
47	Paracingulate sulcus posterior RH	SalVentAttnA	0.625352025	0.157670598	0.216721369
48	Middle frontal gyrus mid-anterior LH	SalVentAttnB	0.635662245	0.144922237	0.09563041
49	Middle frontal gyrus posterior lateral RH	ContB	0.565873584	0.223992114	0.105685969
50	Intraparietal sulcus superior LH	ContB	0.473332146	0.077996236	0.223384856
51	Ventromedial prefrontal cortex	DefaultA	0.736777448	0.122046733	0.141181985
52	Lingual gyrus posterior	VisCent	0.686312324	0.143436391	0.149460501
53	Insula antero-superior RH	SalVentAttnA	0.780743556	0.089289092	0.129465669
54	Caudate superior	No network found	0.260873519	0.684551548	0.054563936
55	Parieto-occipital sulcus mid-posterior	ContC	0.517021905	0.411413131	0.071522748
56	Globus pallidus anterior	SalVentAttnB	0.487870074	0.511397716	0.000732913
57	Superior longitudinal fasciculus I anterior LH	No network found	0.043086854	0.956668649	0.000252436
58	Superior longitudinal fasciculus I anterior RH	No network found	0.025692012	0.974205724	9.74513E-05
59	Precentral sulcus mid-inferior LH	ContA	0.660247763	0.263557722	0.075350134
60	Cerebellum VI superior medial	No network found	0.821612746	0.020712592	0.152959677
61	Postcentral sulcus inferior RH	DorsAttnB	0.329287471	0.653727224	0.016968066
62	Cuneus middle posterior RH	VisPeri	0.479114605	0.456160883	0.062404866
63	Cerebrospinal fluid (between middle frontal gyrus superior and skull LH)	ContB	0.53926698	0.048796793	0.14361224
64	Paracingulate gyrus posterior LH	SomMotA	0.633962149	0.24117342	0.124873363
65	Precentral gyrus mid-superior	DorsAttnB	0.464979086	0.080382549	0.193265162
66	Superior temporal gyrus mid-anterior LH	TempPar	0.797321851	0.08963793	0.108515935
67	Fusiform gyrus anterior	DefaultC	0.788815609	0.144645125	0.066550641

Table S1. Description of the 512 Regions of Interest as described by Yeo Dictionary Atlas.

68	Superior frontal gyrus medial anterior	DefaultA	0.666300246	0.09669621	0.229399291
69	Superior frontal gyrus mid-posterior	SalVentAttnB	0.537307017	0.412071667	0.045577386
70	Cerebellum IX	No network found	0.723533867	0.188568602	0.087908178
71	Thalamus posterior	No network found	0.736321993	0.259094426	0.004576953
72	Globus pallidus RH	No network found	0.161074743	0.838355039	0.000577517
73	Postcentral sulcus RH	SomMotA	0.462255553	0.464206743	0.071026019
74	Thalamus superior	No network found	0.525033424	0.474915902	6.08077E-05
75	Superior occipital sulcus superior LH	DorsAttnA	0.537839875	0.320162757	0.133609391
76	Supramarginal gyrus posterior LH	SalVentAttnB	0.622492608	0.225945156	0.087835167
77	Gyrus rectus	LimbicB	0.580008258	0.297691083	0.122012854
78	Anterior occipital sulcus RH	VisCent	0.636495914	0.328158656	0.035159848
79	Lateral fissure anterior	TempPar	0.567162503	0.02014067	0.346891757
80	Corona radiata posterior LH	No network found	0.016688985	0.983266103	3.05328E-05
81	Cerebellum V anterior	DefaultC	0.840033957	0.011744873	0.148230107
82	Parieto-occipital sulcus mid-anterior	DefaultC	0.775895562	0.121462843	0.102652391
83	Hippocampus anterior	No network found	0.749665284	0.130729133	0.119616404
84	Superior frontal gyrus mid-anterior LH	DefaultB	0.558752335	0.387134455	0.050689266
85	Cerebellum Crus II anterior LH	No network found	0.711262947	0.258648649	0.029549041
86	Intraparietal sulcus middle RH	ContA	0.576446864	0.360686624	0.062805501
87	Parieto-occipital sulcus posterior lateral	VisPeri	0.405713396	0.543828741	0.04654605
88	Occipital pole LH	VisCent	0.489877413	0.433708557	0.059249044
89	Corticospinal tract superior	No network found	0.014549358	0.98544955	4.3066E-06
90	Cingulate gyrus middle superior	SalVentAttnA	0.749980965	0.026498029	0.223525015
91	Parieto-occipital sulcus anterior	DefaultC	0.611236435	0.354115638	0.034635665
92	Cererbrospinal fluid (between callosomarginal sulcus and skull LH)	DorsAttnB	0.460838872	0.086070558	0.254005733
93	Occipitotemporal gyrus posterior RH	DorsAttnA	0.625174391	0.347182662	0.02762415
94	Insula postero-superior lateral LH	SomMotB	0.721810919	0.214064469	0.064137628
95	Superior frontal gyrus posterior LH	DorsAttnB	0.582642877	0.171111177	0.180730983
96	Inferior frontal sulcus middle RH	ContA	0.644326034	0.268616092	0.073188347
97	Postcentral gyrus middle LH	SomMotA	0.536902878	0.193470988	0.170537405
98	Arcuate fasciculus postero-inferior LH	SomMotB	0.060460564	0.939039403	0.000507046
99	Angular gyrus anterior RH	SalVentAttnB	0.620165076	0.20910885	0.088601139
100	Angular sulcus inferior RH	DefaultA	0.529492583	0.429839955	0.039116299
101	Temporal pole LH	DefaultB	0.789354152	0.118720754	0.073152401

Table S1. Description of the 512 Regions of Interest as described by Yeo Dictionary Atlas.

102	Paracentral lobule inferior LH	SomMotA	0.625314369	0.17076462	0.203931935
103	Precentral sulcus medial	SomMotA	0.602816165	0.165753402	0.230163754
104	Dorsomedial prefrontal cortex LH	DefaultB	0.622845274	0.173266113	0.201657319
105	Amygdala inferior	LimbicA	0.916411557	0.026621188	0.05682835
106	Perirhinal cortex	LimbicA	0.607432396	0.354085466	0.03845772
107	Cuneus middle	VisPeri	0.675588928	0.214946411	0.109466267
108	Callosomarginal sulcus middle	SomMotA	0.280221082	0.704689843	0.015093567
109	Lunate sulcus RH	VisCent	0.473091866	0.492074191	0.034235565
110	Putamen superior	No network found	0.106487034	0.893506685	1.27836E-05
111	Putamen LH	No network found	0.434488396	0.56548484	2.37514E-05
112	Frontal pole	DefaultA	0.453391922	0.043064315	0.12413394
113	Forceps minor	No network found	0.004884799	0.99508163	2.95797E-05
114	Superior temporal gyrus medial anterior	LimbicA	0.624822516	0.039366128	0.333258964
115	Middle frontal gyrus anterior RH	SalVentAttnB	0.65848741	0.216017754	0.094186597
116	Planum polare superior	SomMotB	0.644296995	0.02591727	0.329795644
117	Dorsomedial prefrontal cortex posterior	ContB	0.679837561	0.033259991	0.286467035
118	Superior frontal gyrus postero-superior LH	SalVentAttnB	0.637763713	0.136018207	0.104922519
119	Supramarginal gyrus postero-superior RH	ContB	0.503873108	0.442755337	0.045479808
120	Amygdala	No network found	0.768932801	0.09650655	0.134551895
121	Cerebellum VI medial RH	No network found	0.905453887	0.070978712	0.023569618
122	Lingual gyrus medial	VisPeri	0.695510114	0.071360516	0.233127436
123	Cuneus anterior RH	VisPeri	0.471080358	0.483247036	0.045675666
124	Superior frontal sulcus middle LH	DefaultA	0.622838102	0.269168554	0.106088002
125	Cerebellum IX inferior	No network found	0.576667993	0.359399909	0.063927542
126	Cingulum anterior	SalVentAttnB	0.142103073	0.856511394	0.001386899
127	Inferior temporal sulcus mid-posterior RH	ContA	0.708457862	0.255125417	0.034891921
128	Cerebellum VI superior LH	VisPeri	0.832012316	0.014243832	0.153622612
129	Optic radiation LH	No network found	0.041821495	0.958049539	0.000133167
130	Corpus callosum genu inferior	No network found	0.319354966	0.423883713	0.256768459
131	Precuneus mid-superior LH	ContC	0.612253926	0.279596225	0.107521593
132	Postcentral gyrus mid-inferior	SomMotB	0.598812665	0.22219784	0.102274194
133	Inferior frontal sulcus posterior LH	ContA	0.389846025	0.59368093	0.016384807
134	Lateral occipital cortex posterior LH	VisCent	0.64845787	0.157302443	0.140446602
135	Angular gyrus mid-posterior RH	DefaultA	0.616136032	0.321525635	0.050216796

Table S1. Description of the 512 Regions of Interest as described by Yeo Dictionary Atlas.

136	Temporal pole	LimbicA	0.724868017	0.227228791	0.04565789
137	Cerebellum Crus II posterior LH	No network found	0.821402467	0.126152325	0.050394775
138	Superior temporal sulcus posterior LH	DorsAttnA	0.390920887	0.59177487	0.0173013
139	Cerebrospinal fluid (between superior precentral gyrus and skull)	SomMotA	0.484140425	0.328254311	0.128389609
140	Lunate sulcus LH	VisCent	0.498083552	0.468896953	0.032781001
141	Cerebellum Crus I medial RH	No network found	0.844054174	0.121607788	0.034089635
142	Posterior cingulate cortex LH	DefaultA	0.713457441	0.198045986	0.088497433
143	Precuneus anterior	SalVentAttnA	0.646250191	0.115127594	0.238635092
144	Intraparietal sulcus middle LH	ContA	0.560574819	0.313107365	0.125153881
145	Lingual gyrus middle	VisPeri	0.731645552	0.093001612	0.175354528
146	Superior temporal gyrus posterior RH	TempPar	0.597701787	0.340359593	0.052556852
147	Lateral orbital cortex	DefaultB	0.803315379	0.124500344	0.060642483
148	Pons antero-superior	No network found	0.41394441	0.310645208	0.167701098
149	Middle frontal sulcus anterior	SalVentAttnB	0.217757473	0.778380036	0.003871644
150	Occipital pole superior LH	VisCent	0.518282064	0.418566204	0.060133774
151	Lingual gyrus mid-posterior LH	VisCent	0.549069954	0.420691437	0.030235156
152	Posterior cingulate cortex superior	ContC	0.74312399	0.097924279	0.158968303
153	Inferior frontal sulcus posterior RH	ContA	0.63225108	0.30416415	0.063112487
154	Cerebrospinal fluid (between angular gyrus and skull LH)	DefaultA	0.518680193	0.03725501	0.18527621
155	Intraparietal sulcus mid-anterior LH	ContA	0.515276947	0.440427281	0.044295404
156	Arcuate fasciculus postero-inferior RH	TempPar	0.221291963	0.774072281	0.004627765
157	Frontal pole lateral RH	ContB	0.5524167	0.071978585	0.122404458
158	Cerebellum VIIIb inferior	No network found	0.870803492	0.108935418	0.018819098
159	Parieto-occipital sulcus middle superior LH	ContC	0.688996462	0.206042526	0.104944591
160	Anterior rami of the lateral fissure RH	SalVentAttnB	0.640547453	0.27708404	0.08072159
161	Cingulate gyrus mid-posterior	SalVentAttnA	0.794944015	0.062322981	0.142738431
162	Superior frontal sulcus posterior LH	ContA	0.574393658	0.358879796	0.065979253
163	Superior longitudinal fasciculus I posterior LH	DorsAttnB	0.286586322	0.686925397	0.026468839
164	Cerebellum IV inferior	No network found	0.695116647	0.274991159	0.029905629
165	Corona radiata superior	No network found	0.010958004	0.989032777	1.00608E-05
166	Insula postero-superior lateral	SomMotB	0.771019483	0.033857556	0.195129309
167	Middle frontal gyrus posterior LH	DefaultB	0.597052186	0.31296777	0.078350635
168	Third ventricle	No network found	0.468140073	0.199944398	0.331917237
169	Precentral gyrus middle LH	DorsAttnB	0.510199511	0.432203025	0.052996151

Table S1. Description of the 512 Regions of Interest as described by Yeo Dictionary Atlas.

170	Callosal sulcus posterior	ContC	0.539374035	0.383349775	0.077281115
171	Angular gyrus superior LH	DefaultA	0.548672909	0.395622028	0.049236803
172	Superior occipital gyrus LH	DorsAttnA	0.621634283	0.242558572	0.123999109
173	Superior longitudinal fasciculus II posterior RH	ContA	0.182129712	0.813457098	0.004411705
174	Superior frontal gyrus anterior	DefaultB	0.365141096	0.606505776	0.02773049
175	Cuneus posterior RH	VisCent	0.394531074	0.577161309	0.028292586
176	Superior parts of central and postcentral sulci	SomMotA	0.469438641	0.331313431	0.184870182
177	Calcarine sulcus mid-anterior	VisPeri	0.500559021	0.397775258	0.101659856
178	Middle temporal gyrus medial LH	SalVentAttnA	0.372035051	0.607886702	0.020071391
179	Occipital pole lateral LH	VisCent	0.529379198	0.340603594	0.109710782
180	Fusiform gyrus middle superior RH	VisCent	0.55023375	0.421681304	0.028086024
181	Cerebrospinal fluid (between superior parietal lobule and skull RH)	DorsAttnB	0.341619188	0.071999056	0.15962381
182	Cerebrospinal fluid (between superior frontal gyrus and skull)	DefaultB	0.441650062	0.011121631	0.170268941
183	Planum temporale	SomMotB	0.404318738	0.581293677	0.014397547
184	Subcentral gyrus RH	SomMotB	0.615896012	0.116821247	0.116896325
185	Middle frontal sulcus anterior LH	SalVentAttnB	0.637435297	0.258905135	0.08247774
186	Anterior cingulate cortex	DefaultA	0.740054665	0.109201072	0.150744499
187	Inferior occipital gyrus	VisCent	0.775145989	0.130783075	0.080130211
188	Cerebrospinal fluid (between superior frontal gyrus lateral RH and skull)	DefaultA	0.463938488	0.020683331	0.121532555
189	Arcuate fasciculus postero-superior	SalVentAttnA	0.424863731	0.544655549	0.03048302
190	Superior frontal sulcus anterior LH	SalVentAttnB	0.602736472	0.322443878	0.071926145
191	Cingulum middle	SalVentAttnA	0.201127986	0.797623097	0.001266023
192	Frontal pole medial	DefaultA	0.604873963	0.216889863	0.148100324
193	Supramarginal gyrus inferior LH	SalVentAttnA	0.662233686	0.166279255	0.104201017
194	Cerebrospinal fluid (between superior cerebellum and limbic lobe)	VisPeri	0.311548304	0.034641434	0.653821166
195	Occipital pole	VisCent	0.519313931	0.365377044	0.083103455
196	Angular gyrus anterior LH	SalVentAttnA	0.608750317	0.313876369	0.074317907
197	Corpus callosum genu	DefaultA	0.386412793	0.584181497	0.029422965
198	Cerebellum VI antero-inferior LH	No network found	0.625185348	0.275201418	0.081351968
199	Precentral gyrus superior RH	SomMotA	0.473325316	0.455738724	0.067106391
200	Occipital pole superior	VisCent	0.534742719	0.2510474	0.143396792
201	Precentral sulcus mid-superior LH	DorsAttnB	0.560423349	0.398230295	0.041246958
202	Superior occipito-frontal fasciculus middle	No network found	0.377339926	0.450746236	0.171919022
203	Middle frontal gyrus mid-posterior RH	ContB	0.59118306	0.312574873	0.074815642

Table S1. Description of the 512 Regions of Interest as described by Yeo Dictionary Atlas.

204	Pons	No network found	0.505629223	0.494302088	5.69098E-05
205	Middle frontal gyrus anterior lateral RH	SalVentAttnB	0.630223317	0.312823069	0.048391195
206	Postcentral sulcus superior RH	DorsAttnB	0.545027976	0.372182163	0.082651428
207	Superior temporal sulcus posterior RH	TempPar	0.674758227	0.271988041	0.053133516
208	Corpus callosum genu superior	No network found	0.194483987	0.480849206	0.324668485
209	Fourth ventricle	No network found	0.479753075	0.170565698	0.349684424
210	Calcarine sulcus middle	No network found	0.156489832	0.80232154	0.041188731
211	Postcentral sulcus superior LH	SomMotA	0.458510409	0.465266243	0.076051745
212	Precentral gyrus superior LH	DorsAttnB	0.457254876	0.481489922	0.058400841
213	Precentral sulcus mid-superior RH	DorsAttnB	0.505970598	0.455880612	0.036175894
214	Intraparietal sulcus anterior RH	ContA	0.620849635	0.178271975	0.183721515
215	Superior temporal gyrus medial anterior RH	SalVentAttnA	0.542585028	0.408834462	0.048575288
216	Precuneus postero-inferior RH	ContC	0.53820746	0.420305448	0.041492176
217	Parieto-occipital sulcus postero-superior	VisPeri	0.60072283	0.188189642	0.197963832
218	Cerebellum III inferior	No network found	0.781618546	0.176988511	0.041388109
219	Superior parietal lobule medial RH	DorsAttnA	0.423826172	0.503108215	0.06898357
220	Anterior cingulate cortex superior	SalVentAttnB	0.777142427	0.040976857	0.181873646
221	Middle frontal gyrus postero-inferior LH	ContA	0.57334564	0.358578283	0.066708608
222	Inferior longitudinal fasciculus	No network found	0.035695782	0.96149895	0.002789433
223	Cingulate sulcus middle	SalVentAttnB	0.725152466	0.127769975	0.147081697
224	Subthalamic nuclei	No network found	0.333150441	0.648127571	0.018712905
225	Caudate inferior	No network found	0.624429116	0.104595209	0.270988856
226	Parietal operculum posterior RH	SomMotB	0.754621264	0.129452342	0.1159365
227	Collateral sulcus posterior RH	VisCent	0.338968215	0.651709386	0.00932978
228	Cuneus postero-superior	VisPeri	0.56748777	0.162756865	0.165526647
229	Paracentral sulcus	SomMotA	0.252404884	0.738175251	0.009388093
230	Middle temporal gyrus middle posterior LH	DefaultB	0.625150988	0.343566425	0.029371858
231	Inferior temporal sulcus posterior LH	DorsAttnA	0.637512946	0.332299989	0.030155777
232	Occipital pole lateral RH	VisCent	0.580627683	0.379916011	0.037452932
233	Medulla oblongata	No network found	0.525507912	0.398753242	0.043887778
234	Cerebellum Crus II posterior	No network found	0.825446441	0.142349948	0.028856384
235	Superior temporal sulcus middle-superior RH	TempPar	0.640539216	0.306527428	0.052754168
236	Anterior limb of internal capsule	No network found	0.753061704	0.240068755	0.00686761
237	Middle temporal gyrus mid-posterior RH	TempPar	0.766923508	0.144863195	0.079338544

Table S1. Description of the 512 Regions of Interest as described by Yeo Dictionary Atlas.

238	Lateral occipital cortex superior LH	DefaultC	0.583192684	0.176887106	0.146919957
239	Pars triangularis RH	DefaultB	0.597922121	0.153743625	0.108418502
240	Cuneus superior	VisPeri	0.633900962	0.23650154	0.127770179
241	Superior parietal lobule postero-inferior posterior	ContC	0.441589393	0.484346497	0.073319612
242	Superior parietal sulcus LH	DorsAttnA	0.466008601	0.444014065	0.085146092
243	Middle temporal gyrus posterior LH	DorsAttnB	0.681285925	0.095887092	0.149399749
244	Precuneus antero-inferior	ContC	0.601903391	0.33930396	0.058801456
245	Cerebrospinal fluid (between middle frontal gyrus posterior and skull RH)	ContB	0.524480819	0.053683107	0.149132816
246	Superior occipital sulcus middle RH	ContA	0.534883963	0.386168535	0.074577752
247	Supramarginal gyrus anterior RH	DorsAttnB	0.64451108	0.222755773	0.109897077
248	Posterior cingulate cortex inferior	DefaultA	0.76071988	0.073853354	0.165430988
249	Lateral fissure anterior LH	SalVentAttnB	0.768793825	0.035374383	0.192369148
250	Retrosplenial cortex superior	DefaultC	0.520055793	0.23754351	0.242385108
251	Middle frontal gyrus mid-posterior LH	DefaultB	0.564606543	0.359861499	0.062663221
252	Superior temporal gyrus superior RH	SomMotB	0.690753646	0.238337103	0.057367534
253	Precuneus middle	ContC	0.693898664	0.101790138	0.204302635
254	Subsplenial area	No network found	0.343445459	0.093901596	0.562650257
255	Inferior temporal sulcus posterior RH	DorsAttnB	0.539322726	0.432393193	0.027853034
256	Cingulum mid-anterior	SalVentAttnB	0.353252584	0.641246094	0.005511908
257	Cerebellum VI antero-superior LH	No network found	0.847831307	0.098944449	0.052830688
258	Superior parietal lobule LH	DorsAttnB	0.502728145	0.277434145	0.189807222
259	Cerebellum III superior	No network found	0.501915807	0.044524499	0.453570397
260	Cerebellum VI posterior	No network found	0.856205297	0.083305409	0.059323385
261	Caudate	No network found	0.617853621	0.081409434	0.300743811
262	Parahippocampal gyrus anterior	DefaultC	0.758536611	0.192115962	0.049361862
263	Central operculum posterior RH	SomMotB	0.63207677	0.322972516	0.044968601
264	Middle temporal gyrus mid-anterior LH	DefaultB	0.623726122	0.34647429	0.028554083
265	Fusiform gyrus posterior RH	VisCent	0.624506347	0.330741877	0.044733658
266	Frontal operculum RH	SalVentAttnB	0.53090118	0.440455667	0.028634054
267	Superior frontal gyrus middle LH	DefaultB	0.625754483	0.311267135	0.052687445
268	Middle temporal gyrus posterior RH	DorsAttnA	0.651108644	0.297351179	0.050934159
269	Precentral gyrus inferior LH	SomMotB	0.577634235	0.3377177	0.069410266
270	Hippocampus	DefaultC	0.573093573	0.144210556	0.275500568
271	Paracingulate gyrus mid-anterior LH	DefaultA	0.64849881	0.257834122	0.093657647

Table S1. Description of the 512 Regions of Interest as described by Yeo Dictionary Atlas.

272	Cerebellum VIIIb medial	No network found	0.659579993	0.293361251	0.047064221
273	Central sulcus mid-superior RH	SomMotA	0.365960016	0.607503408	0.026528893
274	Cerebellum VIIIb anterior	No network found	0.824235076	0.155813441	0.017050911
275	Cerebrospinal fluid (between superior frontal gyrus superior and skull)	SalVentAttnA	0.411655197	0.045348915	0.100928854
276	Superior parietal lobule postero-superior LH	DorsAttnA	0.510139207	0.30264171	0.130880774
277	Superior frontal sulcus mid-posterior RH	ContB	0.643296761	0.248079615	0.106559369
278	Middle occipital sulcus LH	VisCent	0.408194206	0.56844097	0.023103755
279	Internal capsule posterior RH	No network found	0.198080197	0.676765371	0.12516548
280	Occipitotemporal gyrus posterior LH	VisCent	0.619977766	0.357541864	0.022458456
281	Inferior occipital sulcus	VisCent	0.46226561	0.522065473	0.015529912
282	Middle frontal gyrus anterior LH	SalVentAttnB	0.492912594	0.472996732	0.032747551
283	Lateral occipital cortex antero-superior RH	DorsAttnA	0.701232549	0.22146905	0.073674791
284	Cerebellum VI superior lateral	VisCent	0.783960378	0.061951203	0.152941194
285	Cerebral peduncles	No network found	0.699195486	0.15872819	0.142079938
286	Occipital pole inferior	VisCent	0.575457048	0.3236235	0.08003073
287	Ventromedial prefrontal cortex LH	DefaultA	0.650259377	0.220899957	0.128841435
288	Calcarine sulcus anterior	DefaultC	0.754724659	0.088834561	0.156451826
289	Cerebellum Crus I posterior RH	No network found	0.833139876	0.111330248	0.051449972
290	Precentral sulcus mid-inferior RH	ContA	0.668325797	0.216221811	0.104288157
291	Dorsomedial prefrontal cortex anterior	DefaultB	0.661640034	0.13334417	0.20099571
292	Inferior temporal sulcus middle RH	ContB	0.473455514	0.510722468	0.015263413
293	Heschl’s gyrus LH	SomMotB	0.7391669	0.077014877	0.182796603
294	Middle frontal gyrus posterior RH	ContB	0.504717	0.432660754	0.055928485
295	Parietal operculum LH	SomMotB	0.59559672	0.323582962	0.080834291
296	Calcarine cortex mid-posterior LH	VisPeri	0.642014159	0.268351546	0.089635298
297	Postcentral sulcus superior	SomMotA	0.425468707	0.450408469	0.114279432
298	Thalamus middle	No network found	0.728322953	0.248679761	0.022992915
299	Superior frontal gyrus medial middle	ContB	0.650530902	0.087948301	0.251155765
300	Inferior frontal gyrus posterior LH	ContA	0.629018289	0.294093671	0.072840769
301	Parieto-occipital sulcus middle inferior LH	VisPeri	0.444641459	0.512286705	0.043035666
302	Subparietal sulcus LH	DefaultA	0.618259356	0.320517485	0.061220754
303	Internal capsule anterior limb RH	No network found	0.417167951	0.580305377	0.002523324
304	Precuneus mid-posterior	ContC	0.634463187	0.230614303	0.134156413
305	Angular gyrus superior RH	ContB	0.513809282	0.436975522	0.046391394

Table S1. Description of the 512 Regions of Interest as described by Yeo Dictionary Atlas.

306	Central sulcus middle LH	SomMotA	0.501734653	0.398001715	0.099642147
307	Superior frontal sulcus middle	DefaultA	0.533816483	0.427211871	0.038943837
308	Globus pallidus	No network found	0.244999381	0.754937401	8.10643E-05
309	Central sulcus inferior RH	SomMotB	0.523360626	0.406096989	0.067918873
310	Middle frontal gyrus middle	SalVentAttnB	0.687215356	0.221572784	0.076681116
311	Cerebrospinal fluid (between supramarginal gyrus and skull LH)	DorsAttnB	0.649202703	0.098359854	0.125452934
312	Superior frontal gyrus middle RH	DefaultA	0.637942707	0.264647115	0.092756591
313	Corticospinal tract middle RH	No network found	0.030880591	0.969078185	5.21276E-05
314	Cingulate sulcus posterior RH	SomMotA	0.674769738	0.258082319	0.067153248
315	Lateral occipital cortex superior RH	DefaultC	0.659848276	0.28248753	0.055702343
316	Putamen postero-inferior	No network found	0.631650614	0.354432198	0.013932491
317	Cerebellum horizontal fissure medial	No network found	0.794558156	0.103520563	0.082455245
318	Superior parietal sulcus RH	DorsAttnB	0.504635991	0.328893955	0.156828884
319	Calcarine cortex anterior	VisPeri	0.651357286	0.282331577	0.066325169
320	Precentral sulcus superior	SomMotA	0.44751205	0.51115564	0.040223644
321	Cuneus posterior	VisPeri	0.557901368	0.256836617	0.159048135
322	Precentral sulcus inferior LH	ContA	0.689394095	0.195500166	0.106255169
323	Superior longitudinal fasciculus II posterior LH	DorsAttnB	0.054186596	0.945334951	0.00048586
324	Superior parietal lobule postero-superior RH	DorsAttnA	0.514127051	0.240074573	0.130278247
325	Anterior horizontal ramus of the lateral fissure LH	DefaultB	0.644702889	0.2269983	0.119669885
326	Parieto-occipital sulcus mid-anterior RH	DefaultC	0.356604468	0.624090959	0.019309396
327	Cingulate sulcus mid-anterior RH	SalVentAttnB	0.709585274	0.19347202	0.096951612
328	Caudate LH	No network found	0.663850303	0.32752144	0.008625617
329	Orbital H-shaped sulcus	LimbicB	0.430101458	0.549735836	0.019159055
330	Central operculum LH	SalVentAttnA	0.724682334	0.179694129	0.095588441
331	Parieto-occipital sulcus middle	DefaultC	0.756567461	0.141943076	0.101496304
332	Putamen anterior	No network found	0.739922193	0.259304117	0.000779101
333	Cerebrospinal fluid (between middle frontal gyrus and skull LH)	DefaultB	0.556576981	0.031208853	0.132744493
334	Precentral gyrus mid-superior LH	DorsAttnB	0.53766133	0.108982083	0.155153847
335	Thalamus anterior	No network found	0.346978506	0.653000735	3.64705E-05
336	Posterior cingulate cortex anterior	ContC	0.762422142	0.141883176	0.095691284
337	Superior temporal gyrus posterior LH	TempPar	0.627108456	0.282363027	0.076883693
338	Central sulcus middle	SomMotB	0.417065087	0.537770249	0.045121355
339	Cerebellum IX and cerebellar peduncle	No network found	0.669424975	0.212713618	0.110176244

Table S1. Description of the 512 Regions of Interest as described by Yeo Dictionary Atlas.

340	Calcarine sulcus anterior RH	VisPeri	0.646388291	0.28864727	0.064967206
341	Cerebellum VI medial	No network found	0.559019828	0.438832636	0.002145806
342	Cerebellum V superior	No network found	0.742354547	0.009807345	0.247845137
343	Collateral sulcus anterior	LimbicA	0.697767924	0.262812217	0.038991971
344	Cerebellar peduncles	DefaultC	0.534487121	0.167068863	0.298366888
345	Cerebrospinal fluid (between middle frontal gyrus anterior and skull RH)	SalVentAttnB	0.386216208	0.015279036	0.132015848
346	Cerebellum secondary fissure	No network found	0.829922898	0.127012657	0.04196113
347	Superior occipital gyrus inferior RH	DorsAttnA	0.580849031	0.328452387	0.089410529
348	Central sulcus superior	SomMotA	0.419256136	0.42374453	0.136052831
349	Inferior occipital gyrus anterior LH	ContA	0.798337414	0.097871396	0.094985094
350	Superior parietal lobule posterior	DorsAttnA	0.538790145	0.102112893	0.197173149
351	Superior temporal sulcus mid-posterior LH	TempPar	0.680886786	0.263919321	0.055195462
352	Lateral occipital cortex anterior LH	VisCent	0.570622966	0.39200645	0.037239125
353	Planum polare inferior	SomMotB	0.637437102	0.097732737	0.264764024
354	Paracingulate gyrus anterior LH	DefaultA	0.648713963	0.252445099	0.098844917
355	Paracingulate sulcus posterior	SalVentAttnA	0.60825538	0.312893603	0.078839843
356	Inferior frontal gyrus anterior LH	ContB	0.622729559	0.096846151	0.118054598
357	Angular sulcus inferior LH	DefaultB	0.661949135	0.281876338	0.055868032
358	Subparietal sulcus RH	DefaultA	0.662419714	0.285201967	0.052371275
359	Circular sulcus of the insula anterior	SalVentAttnB	0.745153517	0.174971311	0.079886766
360	Middle temporal gyrus mid-anterior RH	DefaultA	0.784772706	0.108999099	0.101518423
361	Cerebrospinal fluid (between intraparietal sulcus and skull LH)	ContA	0.462381659	0.040588146	0.141427011
362	Precuneus posterior	ContC	0.615980827	0.058670969	0.284713963
363	Inferior occipital gyrus posterior LH	VisCent	0.66075639	0.179278278	0.139174253
364	Lingual gyrus inferior	VisPeri	0.597455117	0.051943624	0.34976984
365	Angular gyrus postero-inferior LH	DefaultA	0.642689754	0.266688972	0.08093647
366	Inferior frontal gyrus anterior RH	SalVentAttnB	0.620740444	0.246733812	0.080758983
367	Paracingulate sulcus mid-posterior	SalVentAttnB	0.631012276	0.157790048	0.211119848
368	Frontomarginal gyrus LH	ContB	0.53137224	0.419130684	0.045010716
369	Middle occipital sulcus RH	VisCent	0.516774831	0.451391568	0.031786488
370	Inferior occipital gyrus RH	VisCent	0.720701001	0.201357093	0.076619191
371	Inferior frontal sulcus anterior RH	ContB	0.456168221	0.519374468	0.021679191
372	Postcentral sulcus inferior LH	DorsAttnB	0.427870142	0.543308937	0.028716545
373	Middle temporal gyrus postero-inferior LH	ContA	0.750597495	0.189869613	0.051308147

Table S1. Description of the 512 Regions of Interest as described by Yeo Dictionary Atlas.

374	Superior parietal sulcus superior	DorsAttnB	0.477290243	0.400377704	0.114218323
375	Cerebellum VI lateral RH	No network found	0.827936357	0.029926958	0.130263126
376	Cerebellum V RH	No network found	0.8067154	0.018473246	0.174373025
377	Internal capsule middle LH	No network found	0.161609967	0.838081223	0.000324275
378	Cingulate sulcus antero-inferior	DefaultA	0.6850836	0.247569108	0.067349287
379	Middle temporal gyrus middle anterior LH	DefaultB	0.570880028	0.400135098	0.028414868
380	Cerebrospinal fluid (between central sulcus and skull LH)	SomMotA	0.381759094	0.112889783	0.14845101
381	Cerebellum VI	No network found	0.33236301	0.667599608	5.05615E-05
382	Cerebrospinal fluid (between central sulcus and skull RH)	SalVentAttnA	0.503997465	0.062280663	0.154706753
383	Corona radiata anterior LH	No network found	0.012738877	0.987111619	0.000155677
384	Superior occipital sulcus superior RH	ContA	0.611188734	0.242836119	0.141088398
385	Intermediate primus of Jensen LH	ContB	0.592482924	0.293059224	0.105852296
386	Lateral occipital cortex antero-superior LH	DorsAttnA	0.595836472	0.346464024	0.056838954
387	Supramarginal gyrus inferior RH	SalVentAttnA	0.694690408	0.137963822	0.111625261
388	Insula antero-superior LH	SalVentAttnB	0.566232171	0.40148635	0.032290211
389	Callosomarginal sulcus mid-inferior	SomMotA	0.739794116	0.048184093	0.212028642
390	Insula postero-inferior	SomMotB	0.65878028	0.233787547	0.107449524
391	Middle temporal gyrus middle RH	DefaultB	0.749879807	0.170965735	0.07038133
392	Retrosplenial cortex inferior	DefaultC	0.25218644	0.002791469	0.74502343
393	Suborbital sulcus	No network found	0.060348741	0.938503025	0.001079245
394	Cingulate sulcus anterior RH	DefaultA	0.592935383	0.361656694	0.045422955
395	Precuneus postero-superior	ContC	0.597592143	0.259232538	0.118290578
396	Superior longitudinal fasciculus I posterior	No network found	0.037551508	0.96203527	0.000429463
397	Middle frontal gyrus anterior lateral LH	SalVentAttnB	0.617441986	0.226190938	0.078022188
398	Cerebrospinal fluid (between callosomarginal sulcus and skull RH)	DorsAttnB	0.465938521	0.091806148	0.197203754
399	Angular sulcus superior RH	ContB	0.654457535	0.255050754	0.072453444
400	Collateral sulcus middle	DefaultC	0.705735284	0.271804551	0.02247535
401	Pars opercularis LH	DefaultB	0.513648886	0.453584537	0.031206148
402	Superior temporal gyrus middle LH	TempPar	0.607989024	0.353509952	0.031382715
403	Occipitotemporal sulcus posterior LH	DorsAttnA	0.532133842	0.449318252	0.018545828
404	Cerebellum Crus I posterior	No network found	0.65387445	0.027150726	0.253631861
405	Lateral fissure posterior limb RH	SomMotB	0.717559914	0.094146473	0.168661628
406	Caudate nucleus	No network found	0.781939593	0.198546804	0.019521266
407	Lateral ventricles posterior horns	No network found	0.295096276	0.334489048	0.370429542

Table S1. Description of the 512 Regions of Interest as described by Yeo Dictionary Atlas.

408	Callosal sulcus mid-posterior	ContC	0.239608103	0.756429635	0.003971385
409	Supramarginal gyrus antero-inferior LH	SalVentAttnA	0.734902223	0.148585591	0.113466496
410	Callosomarginal sulcus superior	SomMotA	0.554547266	0.17647632	0.268946014
411	Angular gyrus antero-inferior LH	TempPar	0.653302326	0.099318528	0.145732425
412	Superior parts of central and postcentral sulci LH	SomMotA	0.464630064	0.402063073	0.126691525
413	Precuneus RH	ContC	0.629216737	0.235625944	0.134070115
414	Superior parts of central and postcentral sulci RH	SomMotA	0.4020748	0.513207623	0.081574895
415	Thalamus LH	No network found	0.3420807	0.656572569	0.001351311
416	Angular gyrus postero-superior LH	DefaultC	0.605252844	0.218319472	0.111969623
417	Paracentral lobule superior	SomMotA	0.551010112	0.204914422	0.24181066
418	Angular gyrus posterior RH	DefaultC	0.691925659	0.216164505	0.082174833
419	Calcarine sulcus posterior medial	VisPeri	0.664175455	0.209709227	0.124406599
420	Paracingulate sulcus posterior LH	SalVentAttnA	0.619086874	0.126849569	0.251048069
421	Thalamus medial	No network found	0.668320389	0.101603079	0.230073416
422	Fusiform gyrus middle inferior RH	VisCent	0.829198368	0.068176169	0.102630526
423	Caudate nucleus tail	No network found	0.416044065	0.15304269	0.430916502
424	Parieto-occipital sulcus postero-inferior RH	VisPeri	0.50916539	0.393844628	0.096770897
425	Cerebellum Crus I superior	VisCent	0.733217352	0.033554848	0.226859016
426	Temporal pole RH	DefaultB	0.768033999	0.140726293	0.067907968
427	Lingual gyrus anterior	VisPeri	0.709260473	0.042417436	0.248332127
428	Cerebrospinal fluid (between postcentral gyrus and skull)	SomMotA	0.259888853	0.068094751	0.084048754
429	Cerebellum Crus II RH	No network found	0.744744197	0.222710955	0.032034283
430	Corpus callosum anterior body	No network found	0.165811343	0.444593939	0.389593453
431	Superior occipital gyrus RH	VisCent	0.696967764	0.201243335	0.09086667
432	Subparietal sulcus inferior LH	DefaultA	0.643890632	0.294685988	0.061419274
433	Intraparietal sulcus mid-posterior RH	DorsAttnA	0.460455357	0.447256783	0.091765312
434	Hippocampus posterior	No network found	0.296411789	0.271511513	0.432076677
435	Cuneus anterior	VisPeri	0.693141513	0.203611906	0.10323641
436	Precuneus antero-superior	DorsAttnB	0.607688617	0.137941025	0.252463421
437	Central sulcus inferior	SomMotB	0.544180606	0.389950198	0.06588618
438	Central sulcus mid-inferior RH	SomMotB	0.510695489	0.411591127	0.077340008
439	Precentral sulcus inferior RH	ContA	0.688999119	0.197358834	0.107619482
440	Superior longitudinal fasciculus I	DorsAttnB	0.05312335	0.946630694	0.000249175
441	Middle frontal gyrus anterior medial RH	SalVentAttnB	0.538547303	0.431870295	0.028998576

Table S1. Description of the 512 Regions of Interest as described by Yeo Dictionary Atlas.

442	Cerebellum Crus I posterior LH	No network found	0.849412137	0.058036711	0.085389362
443	Superior temporal sulcus middle LH	TempPar	0.808436576	0.100397856	0.090620992
444	Cerebrospinal fluid (between intraparietal sulcus and skull RH)	ContB	0.605302742	0.071515579	0.147612006
445	Forceps minor RH	ContB	0.078848799	0.920366519	0.000789332
446	Forceps major	No network found	0.157852287	0.837706165	0.004437784
447	Superior temporal sulcus middle-inferior RH	TempPar	0.637254453	0.311489273	0.05123416
448	Lingual gyrus mid-anterior LH	VisPeri	0.689534795	0.270938456	0.039544802
449	Middle frontal gyrus mid-posterior lateral LH	ContB	0.598892298	0.308317877	0.072132547
450	Cerebellum superior posterior fissure LH	No network found	0.821439005	0.147359869	0.031011076
451	Fusiform gyrus middle RH	VisCent	0.640199593	0.3259268	0.03387467
452	Medulla oblongata superior	No network found	0.527587287	0.35354841	0.118853926
453	Superior occipital gyrus superior RH	DorsAttnA	0.553118425	0.201439604	0.195756496
454	Superior longitudinal fasciculus III posterior RH	No network found	0.020325474	0.979554972	0.000129974
455	Frontomarginal sulcus	LimbicB	0.606495088	0.316538491	0.05519162
456	Superior longitudinal fasciculus III middle RH	No network found	0.093262295	0.889663132	0.017070125
457	Inferior frontal sulcus mid-anterior LH	ContA	0.638708337	0.253342899	0.079257396
458	Cerebrospinal fluid (between superior frontal gyrus middle superior and skull)	DefaultB	0.492972394	0.021097743	0.14128735
459	Central sulcus LH	SomMotB	0.570922966	0.334189912	0.084858147
460	Intraparietal sulcus posterior RH	DorsAttnA	0.475384986	0.450622762	0.070861131
461	Precuneus inferior	DefaultA	0.740305407	0.100671615	0.159028368
462	Heschl's gyrus RH	SomMotB	0.721826532	0.10110165	0.151891374
463	Intraparietal sulcus posterior LH	ContA	0.521556481	0.39708796	0.078642222
464	Lateral ventricles anterior horns	No network found	0.295519085	0.02692429	0.677562317
465	Cerebrospinal fluid (between postcentral sulcus and skull RH)	SomMotA	0.471512226	0.190942743	0.140548513
466	Central sulcus superior LH	SomMotA	0.420234935	0.49210841	0.085594344
467	External capsule middle LH	SalVentAttnA	0.739448773	0.249987937	0.010578426
468	Putamen RH	No network found	0.501976765	0.497826724	0.000199584
469	Cerebrospinal fluid (between superior parietal lobule posterior and skull RH)	DorsAttnA	0.499255539	0.088511002	0.159335486
470	Insula superior	SalVentAttnA	0.80171039	0.118674543	0.074126405
471	Heschl's gyrus posterior RH	SomMotB	0.673933044	0.222210761	0.103847151
472	Optic radiation RH	No network found	0.045413111	0.953175947	0.001402132
473	Anterior parts of occipitotemporal and collateral sulci LH	LimbicA	0.548879512	0.413961493	0.036462353
474	Cingulate sulcus mid-posterior	SalVentAttnA	0.575068577	0.398911891	0.026023868
475	Middle frontal sulcus mid-anterior LH	SalVentAttnB	0.465215083	0.510977739	0.022272309

Table S1. Description of the 512 Regions of Interest as described by Yeo Dictionary Atlas.

476	Ventromedial prefrontal cortex posterior	LimbicB	0.654061239	0.276824702	0.069125901
477	Cerebrospinal fluid (between superior parietal lobule and skull LH)	DorsAttnB	0.383078276	0.06775507	0.13907371
478	Cerebellum VI posterior LH	VisCent	0.870035355	0.041043692	0.08893963
479	Ventromedial prefrontal cortex anterior	LimbicB	0.586667239	0.268300783	0.143981806
480	Putamen postero-superior	No network found	0.526602683	0.473234967	0.000174243
481	Cerebellum VI LH	No network found	0.889806951	0.096225436	0.013976184
482	Cerebellum Crus I superior RH	No network found	0.846046892	0.04144433	0.110223028
483	Superior longitudinal fasciculus III anterior RH	No network found	0.01178538	0.987998279	0.000214056
484	Midbrain inferior	No network found	0.579708897	0.381526466	0.038767644
485	Postcentral gyrus middle RH	DorsAttnB	0.434808411	0.05836386	0.21657899
486	Paracingulate sulcus mid-posterior RH	ContB	0.545644231	0.387044639	0.066861598
487	Fusiform gyrus posterior LH	VisCent	0.558699849	0.412256164	0.029052176
488	Hippocampus middle	No network found	0.617027966	0.331789855	0.051197698
489	Corpus callosum rostrum	LimbicB	0.63860116	0.274693367	0.085747477
490	Orbitofrontal cortex	LimbicB	0.617487693	0.342877073	0.039377708
491	Cerebellum Crus II medial RH	No network found	0.630457316	0.361532948	0.008016378
492	Cerebellum IV superior	No network found	0.875831066	0.072096838	0.05205553
493	Cingulate sulcus posterior LH	SomMotA	0.688177236	0.257625095	0.054196739
494	Superior parietal lobule superior	DorsAttnB	0.581831956	0.232739876	0.17243137
495	Lingual gyrus middle RH	VisPeri	0.540080688	0.4364065	0.023518656
496	Corpus callosum isthmus posterior	ContC	0.058457628	0.941126294	0.000417149
497	Precuneus superior	ContC	0.53262401	0.029299429	0.270372264
498	Inferior occipital gyrus posterior RH	VisCent	0.63869787	0.292218296	0.065387677
499	Callosomarginal sulcus inferior	SalVentAttnA	0.602679428	0.356261625	0.041069823
500	Superior frontal sulcus posterior RH	DorsAttnB	0.615499124	0.264516355	0.116286209
501	Inferior fronto-occipital fasciculus posterior LH	No network found	0.214325734	0.605483007	0.180191495
502	Central sulcus mid-superior	SomMotA	0.470216358	0.407373207	0.122328576
503	Cerebrospinal fluid (between inferior frontal sulcus and skull LH)	ContA	0.524210152	0.052491901	0.172495322
504	Cerebellum Crus I anterior LH	No network found	0.681471888	0.304613043	0.013364418
505	Central operculum anterior RH	SomMotB	0.342846302	0.644569071	0.012590637
506	Corpus callosum splenium	No network found	0.174880943	0.442820413	0.382315374
507	Superior occipital sulcus RH	DorsAttnA	0.494671495	0.458875999	0.046466755
508	Superior temporal gyrus superior LH	SomMotB	0.73042931	0.046016168	0.178493726
509	Middle temporal gyrus mid-posterior LH	TempPar	0.691444654	0.205172354	0.078883626

Table S1. Description of the 512 Regions of Interest as described by Yeo Dictionary Atlas.

510	Insula postero-inferior RH	SalVentAttnA	0.8388826	0.139671923	0.021460774
511	Parieto-occipital sulcus middle LH	ContC	0.742030125	0.087003424	0.170947909
512	Precentral sulcus superior RH	DorsAttnB	0.622743322	0.314314771	0.062787499
